# Pelvic insufficiency fractures after chemoradiotherapy for cervical cancer: incidence, long-term outcomes, and survivorship management

**DOI:** 10.1093/oncolo/oyag427

**Published:** 2026-10-22

**Authors:** Akram Saad, Jeffrey Goldstein, Ronza Atamneh, Limor Helpman, Ronnie Shapira, Omer Berner, Ofir Morag, Oded Jacobi, Dana Oren, Hanifa J Abu-Toamih Atamni, Reut Mordechai, Itay Goor Aryeh, Alexandra Taylor, Shira Peleg Hasson, Shira Felder

**Affiliations:** Gray Faculty of Medical and Health Sciences, Tel Aviv University, Tel Aviv, 6997801, Israel; Department of Medical Oncology, Jusidman Cancer Center, Sheba Medical Center, Tel Hashomer, 5265601, Israel; Department of Radiation Oncology, Tel Aviv Sourasky Medical Center, Tel Aviv, 6423906, Israel; Gray Faculty of Medical and Health Sciences, Tel Aviv University, Tel Aviv, 6997801, Israel; Gray Faculty of Medical and Health Sciences, Tel Aviv University, Tel Aviv, 6997801, Israel; Gynecologic Oncology Unit, Sheba Medical Center, Tel Hashomer, 5265601, Israel; Gray Faculty of Medical and Health Sciences, Tel Aviv University, Tel Aviv, 6997801, Israel; Department of Medical Oncology, Jusidman Cancer Center, Sheba Medical Center, Tel Hashomer, 5265601, Israel; Department of Radiation Oncology, Jusidman Cancer Center, Sheba Medical Center, Tel Hashomer, 5265601, Israel; Gray Faculty of Medical and Health Sciences, Tel Aviv University, Tel Aviv, 6997801, Israel; Department of Medical Oncology, Jusidman Cancer Center, Sheba Medical Center, Tel Hashomer, 5265601, Israel; Gray Faculty of Medical and Health Sciences, Tel Aviv University, Tel Aviv, 6997801, Israel; Department of Medical Oncology, Jusidman Cancer Center, Sheba Medical Center, Tel Hashomer, 5265601, Israel; Gray Faculty of Medical and Health Sciences, Tel Aviv University, Tel Aviv, 6997801, Israel; Department of Medical Oncology, Jusidman Cancer Center, Sheba Medical Center, Tel Hashomer, 5265601, Israel; Clinical Research Laboratory, Meir Medical Center, Kfar Saba, 4428164, Israel; Gray Faculty of Medical and Health Sciences, Tel Aviv University, Tel Aviv, 6997801, Israel; Institute of Pain, Sheba Medical Center, Tel Hashomer, 5265601, Israel; Gray Faculty of Medical and Health Sciences, Tel Aviv University, Tel Aviv, 6997801, Israel; Institute of Pain, Sheba Medical Center, Tel Hashomer, 5265601, Israel; Department of Clinical Oncology, The Royal Marsden Foundation Trust, London, SW3 6JJ, United Kingdom; Gray Faculty of Medical and Health Sciences, Tel Aviv University, Tel Aviv, 6997801, Israel; Department of Medical Oncology, Tel Aviv Sourasky Medical Center, Tel Aviv, 6423906, Israel; Gray Faculty of Medical and Health Sciences, Tel Aviv University, Tel Aviv, 6997801, Israel; Department of Radiation Oncology, Jusidman Cancer Center, Sheba Medical Center, Tel Hashomer, 5265601, Israel

**Keywords:** cervical cancer, pelvic insufficiency fracture, chemoradiotherapy, pelvic radiotherapy, radiation-related toxicity, cancer survivorship

## Abstract

**Background:**

Pelvic insufficiency fractures, a late complication of chemoradiotherapy for cervical cancer, can cause pain and functional impairment. Although most studies have reported incidence rates and risk factors, few have examined clinical presentation, long-term symptom burden, or the impact on survivorship.

**Methods:**

We conducted a retrospective study of patients with cervical cancer treated with chemoradiotherapy from 2018 to 2024. We extracted clinical characteristics, treatment details, dosimetric parameters, imaging studies documenting pelvic insufficiency fractures, and clinical outcomes from the electronic medical record. We used the Kaplan–Meier method to estimate the probability of pelvic insufficiency fractures and univariate Cox regression to assess clinical and dosimetric factors associated with their development.

**Results:**

The cohort comprised 153 patients (median follow-up: 41.4 months). After chemoradiotherapy, 18 patients (11.8%) developed radiologically confirmed pelvic insufficiency fractures (median time to diagnosis: 9.5 months; probability of pelvic insufficiency fracture at 24 months: 11.4%). PET-CT and/or MRI showed that most pelvic insufficiency fractures involved the sacrum. No statistically significant clinical or dosimetric predictors of pelvic insufficiency fractures were identified. All symptomatic patients (*n* = 15, 83%) required referral to pain management services (median observed duration of symptoms: 37 months). No symptomatic patient achieved documented complete symptom resolution during the available follow-up.

**Conclusions:**

Most pelvic insufficiency fractures presented with new-onset pelvic or sacral pain within 2 years of completing chemoradiotherapy. The lack of clinical or dosimetric predictors suggests a multifactorial etiology of fracture risk. Persistent pain, despite multimodal supportive care, led to long-term morbidity. These findings underscore the need for prospective studies to develop evidence-based preventive strategies, survivorship assessments, and management pathways.

## Graphical abstract

**Graphical Abstract:**
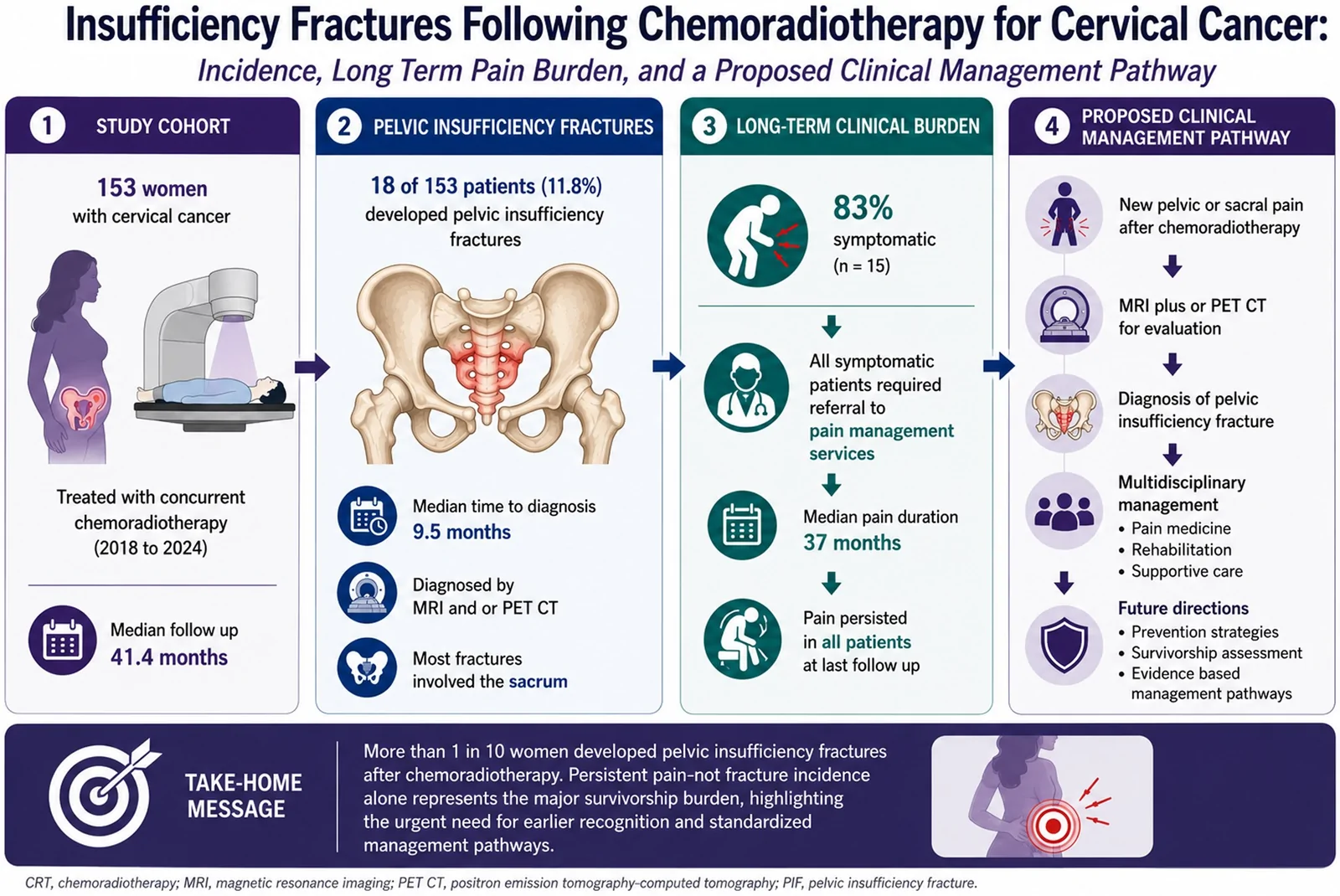

Implications for practice Pelvic insufficiency fractures affect approximately 12% of patients after chemoradiotherapy for cervical cancer and typically develop within 2 years of treatment. In this cohort, no clinical or radiation-dose factors reliably predicted risk of developing a fracture, suggesting that multiple factors influence risk. Because symptomatic fractures often cause persistent pain and substantial disability, new pelvic or lower back pain in survivors should prompt consideration of this diagnosis and appropriate imaging. Survivorship care should include assessment of pain, mobility, and bone health. Prospective studies are needed to develop effective strategies for the prevention, early detection, and management of treatment-related pelvic fractures.

## Introduction

Cervical cancer remains a major global health burden, with persistent geographic disparities despite advances in screening, HPV vaccination, and multimodal treatment.^1^ For patients with locally advanced disease, concurrent chemoradiotherapy followed by brachytherapy remains the backbone of curative treatment, as reflected in current guidelines.^2^^,^^3^ More recently, incorporating neoadjuvant chemotherapy and immunotherapy into primary treatment, as demonstrated in the INTERLACE and KEYNOTE-A18 trials, has further improved oncologic outcomes and may increase the number of long-term survivors who require survivorship-focused care.^4^^,^^5^ Concurrently, advances in image-guided intensity-modulated radiotherapy, volumetric modulated arc therapy, and adaptive brachytherapy have reduced several types of treatment-related toxicity.^6^

As survival improves, the clinical importance of identifying and managing late toxicities that affect mobility, independence, and quality of life has increased. Clinically, pelvic insufficiency fractures often present with pelvic or sacral pain, leading to prolonged morbidity that requires ongoing supportive care. These fractures most commonly involve the sacrum and pelvic ring and result from physiologic stress on structurally weakened bone after chemoradiotherapy.^7^ The reported incidence of pelvic insufficiency fractures after chemoradiotherapy varies widely across studies, from 5% to over 40%, reflecting differences in imaging surveillance, follow-up duration, patient selection, and fracture definitions.^8–12^ Multiple factors may contribute to radiation-related bone injury, including microvascular compromise, impaired bone remodeling, treatment-induced ovarian failure, baseline age- or menopause-related bone loss, body mass index, diabetes mellitus, smoking, low bone mineral density, and sacral radiation exposure. However, findings have been inconsistent across modern intensity-modulated radiotherapy-era cohorts.^13–16^

Most studies report fracture incidence, imaging findings, and risk factors for fracture development, whereas the longitudinal clinical burden after pelvic insufficiency fracture diagnosis remains poorly characterized. Few studies have reported pain duration, supportive care needs, use of pain-mitigating interventions, and long-term symptom outcomes among patients who develop radiation-related pelvic insufficiency fractures.^17^^,^^18^ Furthermore, management strategies for symptomatic pelvic insufficiency fractures vary considerably and may include analgesics, rehabilitation, hyperbaric oxygen therapy, interventional pain procedures, and bone-directed therapies. Evidence evaluating the comparative effectiveness of these approaches remains limited, underscoring the need for standardized, evidence-based treatment pathways.

In this retrospective cohort study of patients treated with modern radiotherapy techniques at a tertiary academic center, we evaluated the incidence, timing, and clinical presentation of pelvic insufficiency fractures and examined clinical and dosimetric factors associated with fracture development. We characterized the clinical course of pelvic insufficiency fractures, including pain burden, supportive care needs, symptom management, and time to symptom resolution. Finally, we addressed the lack of evidence-based clinical pathways to guide the prevention, assessment, and management of radiation-related pelvic insufficiency fractures.

## Materials and methods

### Study design and patient population

We conducted a retrospective cohort study of patients with nonmetastatic cervical cancer, stages IB1-IVA per the Federation International of Gynecological Oncology (FIGO) 2018, treated with definitive or postoperative adjuvant chemoradiotherapy at Sheba Medical Center from 2018 to 2024. Eligibility criteria included age ≥18 years and completion of external beam radiotherapy with concurrent platinum-based chemotherapy, followed by image-guided intracavitary or hybrid intracavitary and/or interstitial brachytherapy when feasible. Exclusion criteria included metastatic bone disease at diagnosis, prior pelvic irradiation, radiotherapy alone, pelvic fractures, incomplete treatment, and inadequate imaging follow-up, defined as the absence of pelvic imaging after chemoradiotherapy. The study flow diagram is shown in Figure S1.

### Radiotherapy and dosimetric assessment

All patients received pelvic external beam radiotherapy and, when clinically indicated, para-aortic external beam radiotherapy, delivered using inverse-planned intensity-modulated radiotherapy or volumetric modulated arc therapy with daily image guidance. We used EMBRACE II contouring recommendations and ESTRO guidelines to plan and deliver external beam radiotherapy and high-dose brachytherapy.^6^^,^^18^ Patients received definitive or postoperative chemoradiotherapy, with simultaneous integrated or sequential nodal boosts delivered per institutional protocols when indicated. Eligible patients underwent image-guided intracavitary or hybrid intracavitary/interstitial brachytherapy.

We converted all radiation doses from brachytherapy to equivalent dose in 2-Gy fractions (EQD2) using an α/β ratio of 10 for tumors and an α/β ratio of 3 for normal tissues.^19^ For dosimetric analysis, we extracted pelvic and sacral dose-volume histogram (DVH) parameters from the external-beam radiotherapy treatment plans. Dosimetric parameters analyzed as continuous variables, included pelvic D50, sacral D50, sacral mean dose, pelvic V30, V40, V50, V52, pelvic V55, and sacral V55.

### Follow-up after chemoradiotherapy

Patients underwent follow-up in accordance with institutional surveillance practices informed by contemporary guidelines.^20^ Surveillance included routine clinical assessment and imaging.^21^ At the physician’s discretion, patients underwent PET-CT or MRI for post-treatment evaluation, surveillance, local assessment, suspected recurrence, equivocal findings, or new pelvic symptoms, as clinically indicated.

### Endpoints and definitions

We defined the primary endpoint as the time from completion of chemoradiotherapy to the first radiologically confirmed pelvic insufficiency fracture. We defined pelvic insufficiency fracture according to the presence of characteristic imaging findings in irradiated pelvic bone in the absence of local tumor involvement. On MRI, diagnostic findings included a discrete fracture line and/or characteristic marrow edema. On PET-CT, diagnosis required a fracture line, sclerosis, or other characteristic structural abnormality on the CT component and considered radiotracer uptake alone insufficient for diagnosis. We identified pelvic insufficiency fractures by reviewing clinical records and imaging studies. Diagnostic confirmation required a discrete fracture line on MRI or PET-CT imaging. Isolated sclerosis without a visible fracture line did not meet the criteria for inclusion as a pelvic insufficiency fracture. We based fracture diagnosis on contemporaneous reports issued by specialist radiologists. Imaging was not centrally re-reviewed for this study.

Patients received post-treatment imaging in accordance with institutional surveillance practices and clinical indications rather than at prespecified study intervals. We recorded the number and modality of post-treatment pelvic imaging examinations, including PET-CT and MRI. We classified imaging performed to evaluate new pelvic, sacral, or lower-back symptoms as symptom-directed imaging.

Secondary endpoints included fracture distribution, imaging modality used for diagnosis, symptomatic presentation, and pain duration among symptomatic patients. We calculated observed symptom duration from the date of fracture diagnosis to the last clinical documentation of fracture-associated symptoms or documented complete symptom resolution, whichever occurred first. Patients with ongoing symptoms at their last assessment were not considered to have achieved symptom resolution. Secondary endpoints also included supportive care required to manage symptomatic patients, such as analgesic use, interventional pain procedures, hyperbaric oxygen therapy, and symptom outcomes during follow-up.

### Statistical analysis

The authors used descriptive statistics to summarize patient, treatment, and dosimetric characteristics; Student’s *t*-test or Mann—Whitney *U* test to compare continuous variables; Chi-square or Fisher’s exact test to compare categorical variables; and the reverse Kaplan–Meier method to estimate follow-up duration and time-to-event. We measured time-to-event from completion of chemoradiotherapy to the date of the first radiologically confirmed pelvic insufficiency fracture, and we censored patients without pelvic insufficiency fracture at the date of last clinical follow-up or death. We used the Kaplan–Meier method to estimate the probability of pelvic insufficiency fractures; the log-rank test to compare groups; and univariate Cox proportional hazards regression to evaluate associations between clinical and dosimetric variables and pelvic insufficiency fracture occurrence. All analyses used 2-sided tests, with *P* ≤ .05 considered statistically significant. All statistical analyses were performed in R version 4.4.2 (R Foundation for Statistical Computing, Vienna, Austria).

### Ethics

The institutional review board (IRB) of Sheba Medical Center approved this study (SMC-5899-19). Given the retrospective design, the IRB waived the requirement for informed consent.

## Results

From 2018 to 2024, we identified 153 patients with cervical cancer who received definitive or adjuvant chemoradiotherapy and met eligibility criteria for this analysis. Table 1 summarizes baseline patient, disease, and treatment characteristics by pelvic insufficiency fracture status. At treatment initiation, the cohort had a median age of 53 years (range, 21-81). Most had stage III disease and received brachytherapy during and after chemoradiotherapy. We observed no significant differences in age, menopausal status, stage, histology, radiation fields, treatment approach, or treatment duration between patients who developed pelvic insufficiency fractures and those who did not.

**Table 1.** Baseline patient and treatment characteristics according to pelvic insufficiency fracture status.

Variable	Overall (*n* = 153)	PIF (*n* = 18)	No PIF (*n* = 135)	*P*-value
**Age at CRT initiation, median (range)**	53 (21-81)	54 (40-72)	53 (21-81)	.7
**Age >50 years**	87 (57%)	11 (61%)	76 (56%)	.7
**Menopausal status**				.8
** Premenopausal**	63 (41%)	7 (39%)	56 (41%)	
** Postmenopausal**	90 (59%)	11 (61%)	79 (59%)	
**Performance status**				.5
** ECOG 0**	130 (85%)	14 (78%)	116 (86%)	
** ECOG 1-2**	23 (15%)	4 (22%)	19 (14%)	
**Smoking history**				.2
** Never smoker**	89 (58%)	8 (44%)	81 (60%)	
** Ever smoker**	64 (42%)	10 (56%)	54 (40%)	
**Chronic diseases**				.3
** None**	76 (50%)	7 (39%)	69 (51%)	
** ≥1 chronic disease**	77 (50%)	11 (61%)	66 (49%)	
**Cannabis use**				.3
** No**	85 (56%)	12 (67%)	73 (54%)	
** Yes**	68 (44%)	6 (33%)	62 (46%)	
**Radiation field**				.8
** Pelvis only**	129 (84%)	15 (83%)	114 (84%)	
** Pelvis + inguinal**	4 (3%)	0	4 (3%)	
** Pelvis + para-aortic**	20 (13%)	3 (17%)	17 (13%)	
**FIGO 2018 stage**				.14
** IB-II**	32 (21%)	3 (17%)	29 (21%)	
** III-IV**	121 (79%)	15 (83%)	106 (79%)	
**Histology**				.7
** Squamous cell carcinoma**	123 (80%)	14 (78%)	109 (81%)	
** Adenocarcinoma/other**	30 (20%)	4 (22%)	26 (19%)	
**Treatment approach**				.15
** Definitive CRT**	147 (96%)	16 (89%)	131 (97%)	
** Adjuvant CRT**	6 (4%)	2 (11%)	4 (3%)	
**Brachytherapy**	145 (95%)	16 (89%)	129 (96%)	.2
**Interstitial brachytherapy**	80 (55%)	11 (69%)	69 (53%)	.2
**Lymph node boost**				.5
** No**	25 (16%)	4 (22%)	21 (16%)	
** Yes**	128 (84%)	14 (78%)	114 (84%)	

Abbreviations: CRT, chemoradiotherapy; ECOG, Eastern Cooperative Oncology Group; FIGO, Federation Internationale de Gynecologie et d’Obstetrique; PIF, pelvic insufficiency fracture.

During follow-up, 18 patients developed radiographically confirmed pelvic insufficiency fractures, for an incidence of 11.8%. Table 2 summarizes clinical characteristics, fracture location, management approaches, and symptom outcomes, with individual patient-level data in Table S1. The median time from completion of chemoradiotherapy to diagnosis of a pelvic insufficiency fracture was 9.5 months (range, 4-65 months), with most fractures occurring within 24 months after treatment completion (Figure 1).

**Figure 1.**
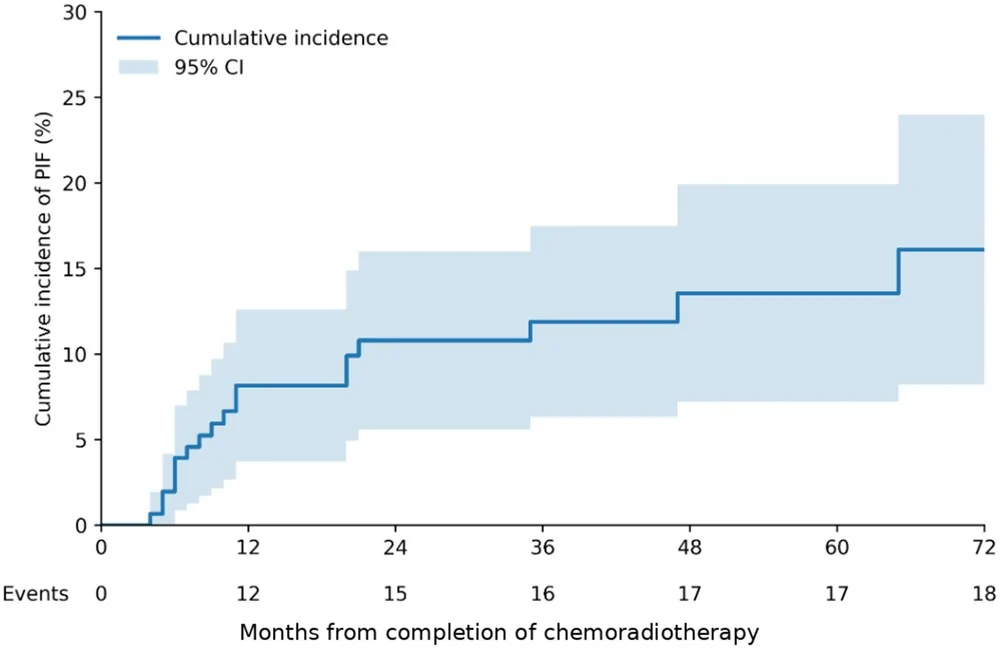
Kaplan–Meier estimated probability of pelvic insufficiency fractures after chemoradiotherapy for cervical cancer. Probability of radiographically confirmed pelvic insufficiency fractures following completion of chemoradiotherapy for cervical cancer.

**Table 2.** Clinical presentation, imaging findings, management, and symptom outcomes of patients with pelvic insufficiency fractures.

Variable	PIF cohort (*n* = 18)
**Symptomatic at diagnosis**	15 (83%)
**Asymptomatic at diagnosis**	3 (17%)
**Median time to PIF diagnosis, months (range)**	9.5 (4-65)
**PET-CT**	10 (56%)
**MRI**	3 (17%)
**MRI and PET-CT**	5 (28%)
**Sacral involvement**	16 (89%)
**Referral to pain management services**	15 (83%)
**Opioid therapy**	7 (39%)
**Cannabis use**	6 (33%)
**Hyperbaric oxygen therapy (HBOT)**	4 (22%)
**Interventional pain procedures (IPP)**	8 (44%)
**Median observed duration of symptoms, months (range)^a^**	37.0 (4.2-94.5)
**Persistent symptoms during follow-up**	9 (50%)
**Partial improvement during follow-up**	6 (33%)
**Asymptomatic throughout follow-up**	3 (17%)

Abbreviations: HBOT, hyperbaric oxygen therapy; MRI, magnetic resonance imaging; PET-CT, positron emission tomography/computed tomography; PIF, pelvic insufficiency fracture.

a Calculated among symptomatic patients.

Among patients diagnosed with pelvic insufficiency fractures, most presented with symptomatic pelvic or sacral pain (*n* = 15, 83%). Asymptomatic fractures detected incidentally during surveillance were uncommon (*n* = 3, 17%). Imaging modalities used for diagnosis included PET-CT (*n* = 10), MRI and PET-CT (*n* = 5), and MRI (*n* = 3). Most fractures involved the sacrum, either in isolation or in combination with adjacent pelvic structures, including the lumbar spine or the iliac bone. Figure 2 presents representative MRI and PET-CT findings of radiation-related pelvic insufficiency fractures.

**Figure 2.**
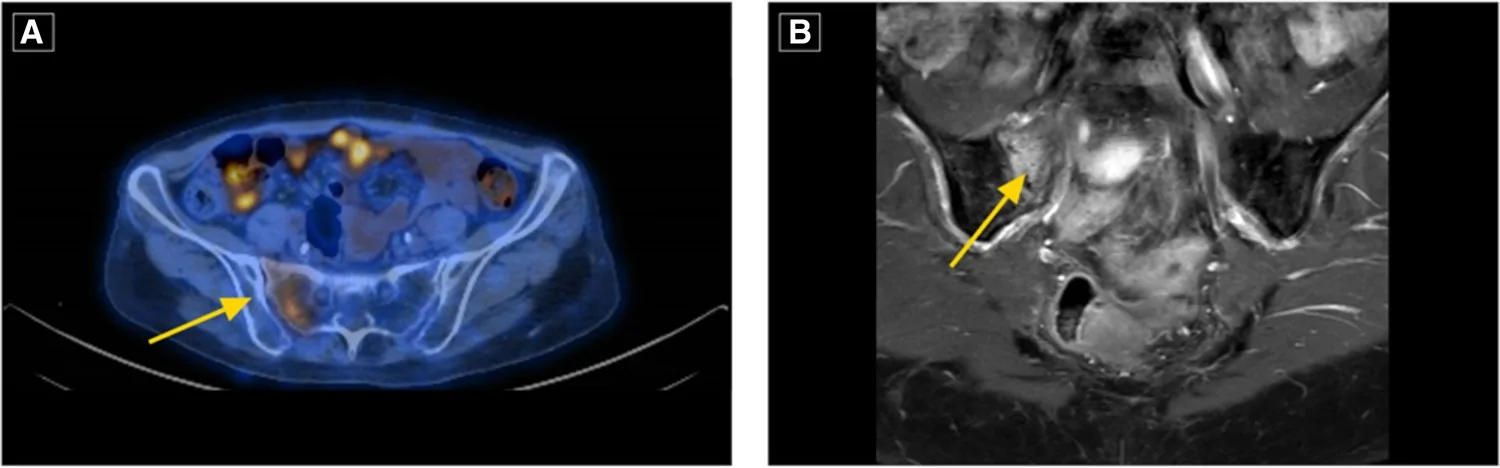
Representative imaging findings of radiation-related pelvic insufficiency fractures. (A) Positron emission tomography-computed tomography (PET-CT) showing focal radiotracer uptake in the right sacrum, corresponding to a pelvic insufficiency fracture identified during evaluation of post-treatment pelvic pain. (B) Coronal T1-weighted magnetic resonance imaging (MRI) showing a right sacral insufficiency fracture with associated bone marrow signal abnormality.

All 15 symptomatic patients required referral to a specialized pain management service. Without an evidence-based clinical pathway, patients received individualized care. Treatments included non-opioid analgesics, cannabis, opioids when clinically indicated, interventional pain procedures (IPP), hyperbaric oxygen therapy, and physical rehabilitation.

Patients had long-lasting symptoms, including pain (median duration, 37.0 months, range, 4.2-94.5 months). Symptom outcomes varied during follow-up. Nine patients (50%) had persistent symptoms with no significant pain relief; 6 (33%) reported partial improvement, and 3 (17%) remained asymptomatic throughout follow-up. No patients reported complete symptom resolution. The prolonged duration of symptoms and the frequent persistence of pain despite supportive management underscore the substantial long-term clinical burden of radiation-related pelvic insufficiency fractures.

Table 3 summarizes dosimetric parameters by pelvic insufficiency fracture status. Pelvic and sacral radiation dose-volume parameters were similar between patients who developed pelvic insufficiency fractures and those who did not. Univariate Cox regression analysis found no significant associations between pelvic insufficiency fracture development and age, menopausal status, radiation field, lymph-node boost, parametrial external beam radiotherapy EQD2, lymph-node external beam radiotherapy EQD2, pelvic D50, sacral D50, pelvic V52, or sacral V55 (Table 4). Definitive compared with adjuvant chemoradiotherapy showed a trend toward association with pelvic insufficiency fracture development but did not reach statistical significance (HR, 0.23; 95% CI, 0.05-1.02; *P* = .054). No clinical or dosimetric variables were significantly associated with pelvic insufficiency fracture development in this cohort.

**Table 3.** Dosimetric parameters according to pelvic insufficiency fracture status.

Variable	Overall (*n* = 153)	PIF (*n* = 18)	No PIF (*n* = 135)	*P*-value
**Parametrial EBRT EQD2, Gy**	49.2 (43.2-77.2)	48.4 (43.2-77.2)	49.2 (43.2-69.6)	.6
**LN EBRT EQD2, Gy**	57.2 (43.2-69.0)	57.2 (43.2-68.0)	57.2 (43.2-69.0)	>.9
**Pelvic D50, Gy**	32.2 (24.4-43.3)	31.9 (26.2-36.2)	32.3 (24.4-43.3)	.2
**Sacrum D50, Gy**	37.3 (26.9-43.6)	36.3 (28.8-40.7)	37.5 (26.9-43.6)	.2
**Sacrum mean dose, Gy**	36.8 (1.6-66.1)	36.0 (27.1-38.6)	36.9 (1.6-66.1)	.043
**Pelvic V30, cc**	282.9 (0-514.4)	285.4 (196-393)	279.1 (0-514.4)	.7
**Pelvic V40, cc**	152.6 (0-341.4)	144.1 (11-234)	151.3 (0-341.4)	.5
**Pelvic V50, cc**	2.0 (0-157.8)	0.6 (0-19.3)	2.1 (0-157.8)	.081
**Pelvic V52, cc**	1.0 (0-73.1)	0.2 (0-13.6)	1.0 (0-73.1)	.074
**Pelvic V55, cc**	0.9 (0-50.6)	0.8 (0-7.4)	0.9 (0-50.6)	>.9
**Sacrum V55, cc**	0.1 (0-35.5)	0.0 (0-3.9)	0.1 (0-35.5)	.7

All values presented as median (range).

Abbreviations: cc, cubic centimeters; D, dose; EBRT, external beam radiotherapy; EQD2, equivalent dose in 2-Gy fractions; Gy, Gray; LN, lymph-node; PIF, pelvic insufficiency fracture; V, volume.

**Table 4.** Selected univariate cox regression analysis for the development of pelvic insufficiency fracture.

Variable	HR (95% CI)	*P*-value
**Age, per year**	1.01 (0.98-1.05)	.58
**Premenopausal vs postmenopausal**	0.84 (0.33-2.18)	.72
**Pelvic + para-aortic field vs pelvic field**	1.43 (0.41-4.95)	.57
**Definitive vs adjuvant treatment**	0.23 (0.05-1.02)	.054
**Lymph-node boost, yes vs no**	0.64 (0.21-1.94)	.43
**Parametrial EBRT EQD2**	0.99 (0.93-1.05)	.77
**Lymph-node EBRT EQD2**	0.99 (0.92-1.06)	.79
**Pelvic D50**	0.89 (0.78-1.03)	.12
**Sacrum D50**	0.91 (0.80-1.03)	.13
**Sacrum mean dose**	0.98 (0.93-1.04)	.57
**Pelvic V52**	0.89 (0.75-1.04)	.15
**Sacrum V55**	0.90 (0.65-1.24)	.51

Abbreviations: CI, confidence interval; D, dose; EBRT, external beam radiotherapy; EQD2, equivalent dose in 2-Gy fractions; HR, hazard ratio; PIF, pelvic insufficiency fracture; V, volume.

## Discussion

### Summary of main results

Among a contemporary cohort of 153 patients with cervical cancer treated with definitive or adjuvant chemoradiotherapy from 2018 to 2024, radiologically confirmed pelvic insufficiency fractures occurred in 18 patients (11.8%). Most fractures involved the sacrum, either alone or in combination with adjacent pelvic structures, and occurred within the first 2 years after treatment completion (median time to diagnosis, 9.5 months). Despite advances in radiotherapy delivery, including intensity-modulated radiotherapy, volumetric modulated arc therapy, image guidance, and MRI-based adaptive brachytherapy, pelvic insufficiency fractures remained a late toxicity observed during cervical cancer survivorship.

Evaluation of new pelvic or sacral pain, rather than routine surveillance imaging, identified most pelvic insufficiency fractures. The fractures detected incidentally in 3 asymptomatic patients did not require clinical intervention. These findings highlight the importance of considering pelvic insufficiency fractures when evaluating new pelvic symptoms after chemoradiotherapy, particularly during the first 2 years after treatment, when fractures were most frequently diagnosed.

### Results in the context of published literature

Reported rates of pelvic insufficiency fractures after pelvic radiotherapy for gynecologic malignancies range from 5% to 40%, reflecting differences in patient populations, follow-up duration, imaging strategies, fracture definitions, and radiotherapy techniques.^8–14^ The 11.8% rate observed in our cohort aligns with contemporary series. The proportion of patients with symptomatic fractures in our cohort (83.3%) was higher than the approximately 61% reported in the meta-analysis by Sapienza et al.^22^ This difference may reflect clinically driven imaging in our cohort, whereby symptomatic patients were more likely to undergo targeted MRI or PET-CT, while asymptomatic fractures may have remained undetected. Differences in imaging schedules, fracture definitions, symptom documentation, and follow-up duration may also have contributed. Studies using routine MRI or PET-CT surveillance have reported higher fracture detection rates, suggesting that imaging intensity influences the identification of asymptomatic or minimally symptomatic fractures.^8^^,^^10^^,^^12^^,^^20^^,^^21^ In contrast, routine follow-up imaging in our cohort identified only 3 asymptomatic pelvic insufficiency fractures, all of which remained clinically silent. These findings suggest that although imaging surveillance may identify abnormalities, most clinically relevant fractures in routine practice present with symptoms that require targeted evaluation.

The timing of fracture development in our cohort aligns with previous reports that most pelvic insufficiency fractures occur within the first 1-2 years after completion of chemoradiotherapy.^9^^,^^11^^,^^14^ The median time to diagnosis of 9.5 months underscores the early survivorship period as a clinically important window for recognizing this complication.

We did not identify statistically robust clinical or dosimetric predictors of pelvic insufficiency fracture in this cohort. Only 18 fracture events occurred, which substantially limited statistical power and the number of variables we could reliably assess. Accordingly, the absence of statistically significant associations should not be interpreted as evidence that established clinical or dosimetric risk factors are unimportant.

Multiple factors, including baseline skeletal health, menopausal status, age-related bone loss, smoking, body mass index, vascular changes, and post-treatment alterations in bone remodeling, contribute to the risk of pelvic insufficiency fractures. Future studies incorporating longitudinal assessment of bone health parameters, including bone mineral density and metabolic markers, may help identify patients at increased risk.

### Clinical impact and management considerations

Most patients with pelvic insufficiency fractures presented with pelvic or sacral pain, prompting additional imaging, supportive care, and referral to specialized pain services. The symptom burden was substantial, with a median observed symptoms duration of 37 months and persistent symptoms in half of patients. The reported symptom duration should be interpreted as the observed follow-up with symptoms rather than the natural time to symptom resolution, because most symptomatic patients continued to report symptoms at their last assessment. Persistent pain following a pelvic insufficiency fracture may not be attributable solely to an unhealed fracture. Potential overlapping contributors include radiation-related fibrosis, neuropathic pain, sacroiliac dysfunction, altered pelvic biomechanics, degenerative musculoskeletal disease, and, less commonly, osteoradionecrosis. In this retrospective study, we did not systematically assess these potential causes, so we could not determine the fractures’ relative contribution to prolonged symptoms. Management strategies included analgesic therapy, IPP, hyperbaric oxygen therapy, and rehabilitation; no patient achieved complete symptom resolution (Table S1).

These findings indicate that symptomatic pelvic insufficiency fracture poses a prolonged survivorship challenge rather than a transient treatment-related event. Previous studies have reported heterogeneous approaches to pelvic insufficiency fracture management, including conservative measures, analgesic therapy, sacroplasty, and other supportive interventions.^23^ However, no standardized clinical guidelines or validated pelvic insufficiency fracture-specific assessment tools exist.^11^ The absence of evidence-based management pathways limits objective assessment of treatment response and the optimization of symptom control.

To address these gaps, prospective use of validated patient-reported outcome measures, including pain intensity scores, functional assessments, and opioid utilization, may enable standardized evaluation of symptom burden and treatment response. Structured multidisciplinary approaches that incorporate bone health assessment, risk-factor modification, symptom-directed management, rehabilitation, and selective procedural interventions warrant prospective evaluation (Figure 3).

**Figure 3.**
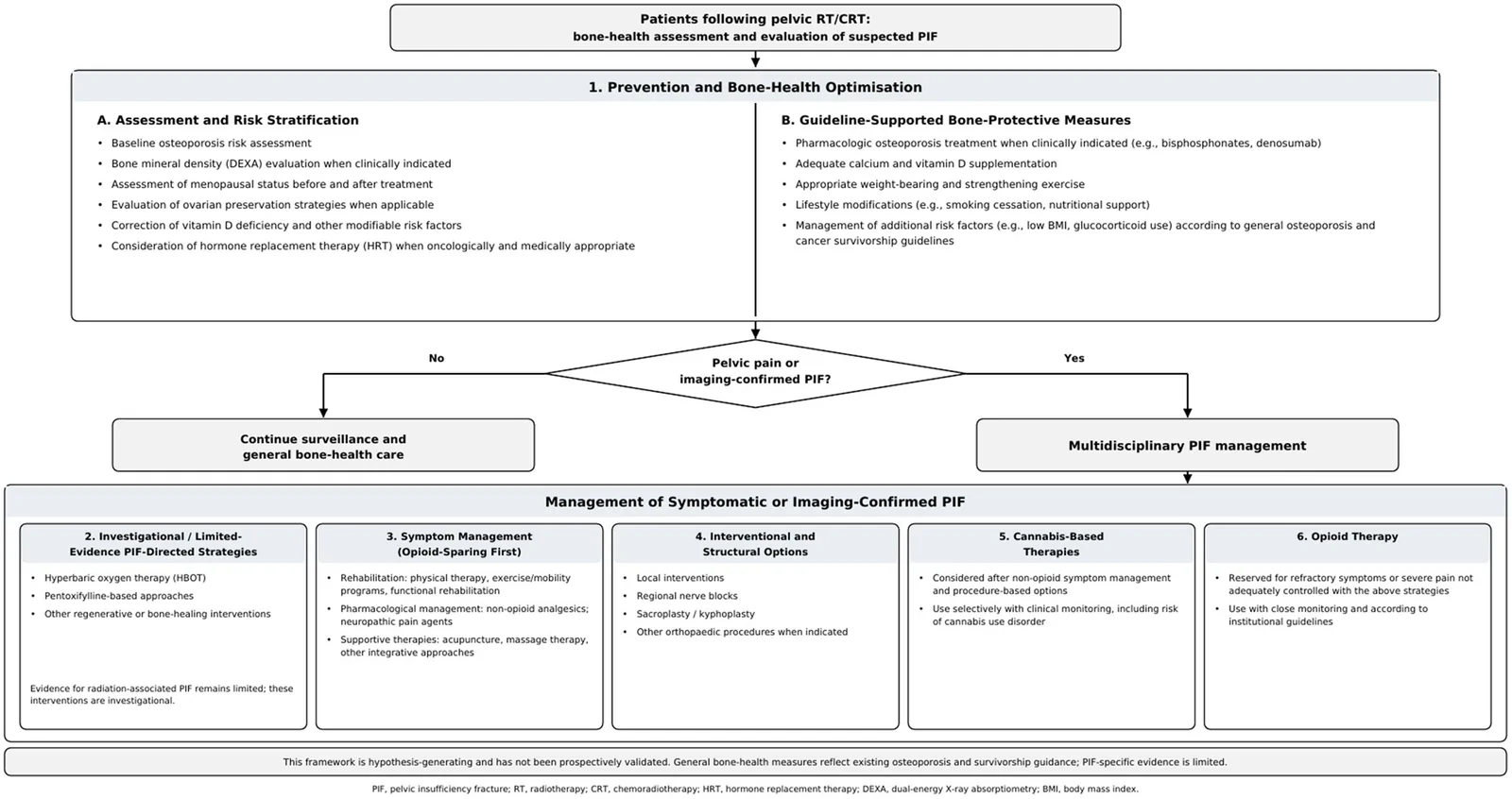
Proposed hypothesis-generating framework for bone health assessment and management after pelvic chemoradiotherapy.

Several interventions, including sacroplasty and hyperbaric oxygen therapy, have shown potential benefit in selected patients with radiation-associated bone or soft-tissue injury; however, evidence supporting their use specifically for radiation-associated pelvic insufficiency fracture after cervical cancer treatment remains limited.^22–26^ Similarly, preventive strategies to preserve bone health require further investigation. Prospective trials evaluating bone-protective interventions during chemoradiotherapy may provide important data on preventing pelvic insufficiency fracture.

Pelvic insufficiency fractures remained clinically relevant despite the uniform use of contemporary intensity-modulated radiotherapy. However, this study did not include a 3-dimensional conformal radiotherapy comparator, so it cannot determine whether IMRT reduced fracture risk. IMRT does not necessarily reduce sacral or pelvic bone exposure^27^ unless these structures are prospectively contoured and incorporated into optimization objectives. Required target coverage, including pelvic nodal regions adjacent to weight-bearing bone, may still result in substantial osseous exposure.

### Strengths and limitations

This study has several strengths, including a contemporary, relatively homogeneous cohort treated with modern radiotherapy; a detailed review of imaging-confirmed fractures; comprehensive clinical characterization; and an evaluation of clinical and dosimetric factors associated with pelvic insufficiency fractures.

The study also has limitations. First, the retrospective, single-institution design introduces potential selection bias and may limit generalizability. Second, the relatively small number of fracture events limited statistical power to identify clinical or dosimetric predictors. Third, risk modeling did not include baseline skeletal health parameters, such as bone mineral density, osteoporosis status, vitamin D levels, and longitudinal bone assessments. HRT exposure was inconsistently recorded and therefore could not be reliably assessed. This is relevant because pelvic radiotherapy may cause premature ovarian insufficiency, and HRT may help preserve bone health in appropriately selected patients. Future studies should prospectively record menopausal status, ovarian function, HRT exposure, and treatment contraindications. Fourth, symptom assessment and treatment response relied on clinical documentation and non-standardized reporting, limiting objective evaluation of symptom trajectories. Fifth, we did not perform imaging on a uniform schedule. Symptomatic patients may have undergone more frequent or targeted imaging, increasing the likelihood of fracture detection, whereas clinically silent fractures may have remained undiagnosed. Sixth, we treated death as a censoring event rather than a competing event in the Kaplan–Meier analysis; this may overestimate fracture probability and absolute fracture incidence. Therefore, interpret the observed proportion of symptomatic fractures in light of potential ascertainment bias. Finally, proposed management frameworks and patient-reported outcome strategies require prospective validation before widespread clinical implementation.

### Implications for practice and future research

In this contemporary cohort, pelvic insufficiency fractures occurred in approximately 1 in 9 patients after chemoradiotherapy for cervical cancer. Diagnosis occurred during early survivorship, prompted by new pelvic or sacral pain. These findings highlight the need to raise clinical awareness of pelvic insufficiency fractures as a potential cause of persistent pelvic symptoms after treatment.

Future research should prioritize prospective identification of risk factors, integration of baseline and longitudinal bone health assessments, development of validated tools to assess symptoms and function, and evaluation of preventive and therapeutic interventions. These efforts may support evidence-based strategies to reduce fracture burden and improve quality of life for cervical cancer survivors.

## Conclusion

In this contemporary cohort, pelvic insufficiency fractures occurred in 18 of 153 patients (11.8%) after chemoradiotherapy for cervical cancer. Most fractures developed within the first 2 years following treatment and were diagnosed after evaluation of new pelvic or sacral pain, whereas routine imaging surveillance identified few asymptomatic fractures. The absence of identifiable clinical or dosimetric predictors suggests that fracture development is multifactorial. Patients with symptomatic pelvic insufficiency fractures experienced substantial prolonged morbidity despite multimodal supportive care. To address the need for evidence-based clinical management pathways, our institution has initiated an investigator-led study evaluating interventions, including bisphosphonate therapy for bone protection in this population,^28^ validated patient-reported outcome measures, and a prospective opioid-sparing clinical management pathway. These findings highlight the importance of prompt imaging evaluation of new pelvic pain during cervical cancer survivorship care and support further prospective investigation of strategies for fracture prevention, early detection, and standardized management of radiation-related pelvic insufficiency fractures.

## Supplementary Material

oyag427_Supplementary_Data

## Data Availability

The data underlying this study are not publicly available because they contain protected patient information. De-identified data may be provided by the corresponding author upon reasonable request, subject to institutional approval and applicable data-protection requirements.
