# Supplementary material for: Pelvic insufficiency fractures after chemoradiotherapy for cervical cancer: incidence, long-term outcomes, and survivorship management
Source: Oncologist. 2026 Oct 22;31(12):oyag427. doi: 10.1093/oncolo/oyag427 (PMC13742277; doi:10.1093/oncolo/oyag427)
Supplement: Supplementary file 1 [file oyag427_supplementary_data.zip › Supplementary Table S1.pdf]

Supplementary Table S1. Patient-Level Clinical Characteristics, Imaging Findings, Management, and Outcomes of Pelvic Insufficiency Fractures

Patient	Time to PIF diagnosis, months	Symptoms	Imaging	Fracture location	Management received	Outcome	Pain duration, months
P1	7	Yes	PET-CT	Sacrum	Pain clinic; opioids; IPP	Persistent symptoms	4.2
P2	20	No	PET-CT	Sacrum + lumbar spine	None	Asymptomatic	NA
P3	10	Yes	PET-CT	Sacrum	Pain clinic; cannabis	Persistent symptoms	51.8
P4	5	Yes	MRI + PET-CT	Sacrum	Pain clinic; opioids; cannabis; IPP	Persistent symptoms	94.5
P5	21	Yes	MRI + PET-CT	Lumbar spine	Pain clinic; IPP	Partial improvement	18.2
P6	6	Yes	MRI + PET-CT	Sacrum	Pain clinic; opioids; cannabis	Persistent symptoms	78.1
P7	5	Yes	PET-CT	Sacrum	Pain clinic; opioids; IPP	Partial improvement	27.2
P8	9	Yes	MRI	Sacrum	Pain clinic; opioids	Persistent symptoms	22.6
P9	47	Yes	PET-CT	Sacrum	Pain clinic; IPP	Partial improvement	51.8
P10	35	Yes	MRI	Sacrum + iliac bone	Pain clinic; cannabis; HBOT; IPP	Partial improvement	48.7
P11	6	Yes	PET-CT	Sacrum	Pain clinic; HBOT; IPP	Partial improvement	49.7
P12	20	Yes	MRI + PET-CT	Sacrum	Pain clinic; opioids; cannabis; HBOT; IPP	Partial improvement	37.0
P13	11	Yes	PET-CT	Sacrum + lumbar spine	Pain clinic; cannabis	Persistent symptoms	30.7
P14	65	No	MRI + PET-CT	Lumbar spine	None	Asymptomatic	NA
P15	4	Yes	PET-CT	Sacrum	Pain clinic; opioids	Persistent symptoms	18.3
P16	11	No	PET-CT	Lumbar spine	None	Asymptomatic	NA

Patient	Time to PIF diagnosis, months	Symptoms	Imaging	Fracture location	Management received	Outcome	Pain duration, months
P17	8	Yes	MRI	Sacrum	Pain clinic; opioids; HBOT; IPP	Persistent symptoms	5.0
P18	6	Yes	PET-CT	Sacrum + lumbar spine	Pain clinic	Persistent symptoms	51.8

Abbreviations: PIF, pelvic insufficiency fracture; PET-CT, positron emission tomography-computed tomography; MRI, magnetic resonance imaging; HBOT, hyperbaric oxygen therapy; NA, not applicable; IPP, interventional pain procedure.
