# Supplementary material for: Pelvic insufficiency fractures after chemoradiotherapy for cervical cancer: incidence, long-term outcomes, and survivorship management
Source: Oncologist. 2026 Oct 22;31(12):oyag427. doi: 10.1093/oncolo/oyag427 (PMC13742277; doi:10.1093/oncolo/oyag427)
Supplement: Supplementary file 1 [file oyag427_supplementary_data.zip › Supplemental_Figure_S1.pdf]

Supplemental Figure S1: study flow diagram

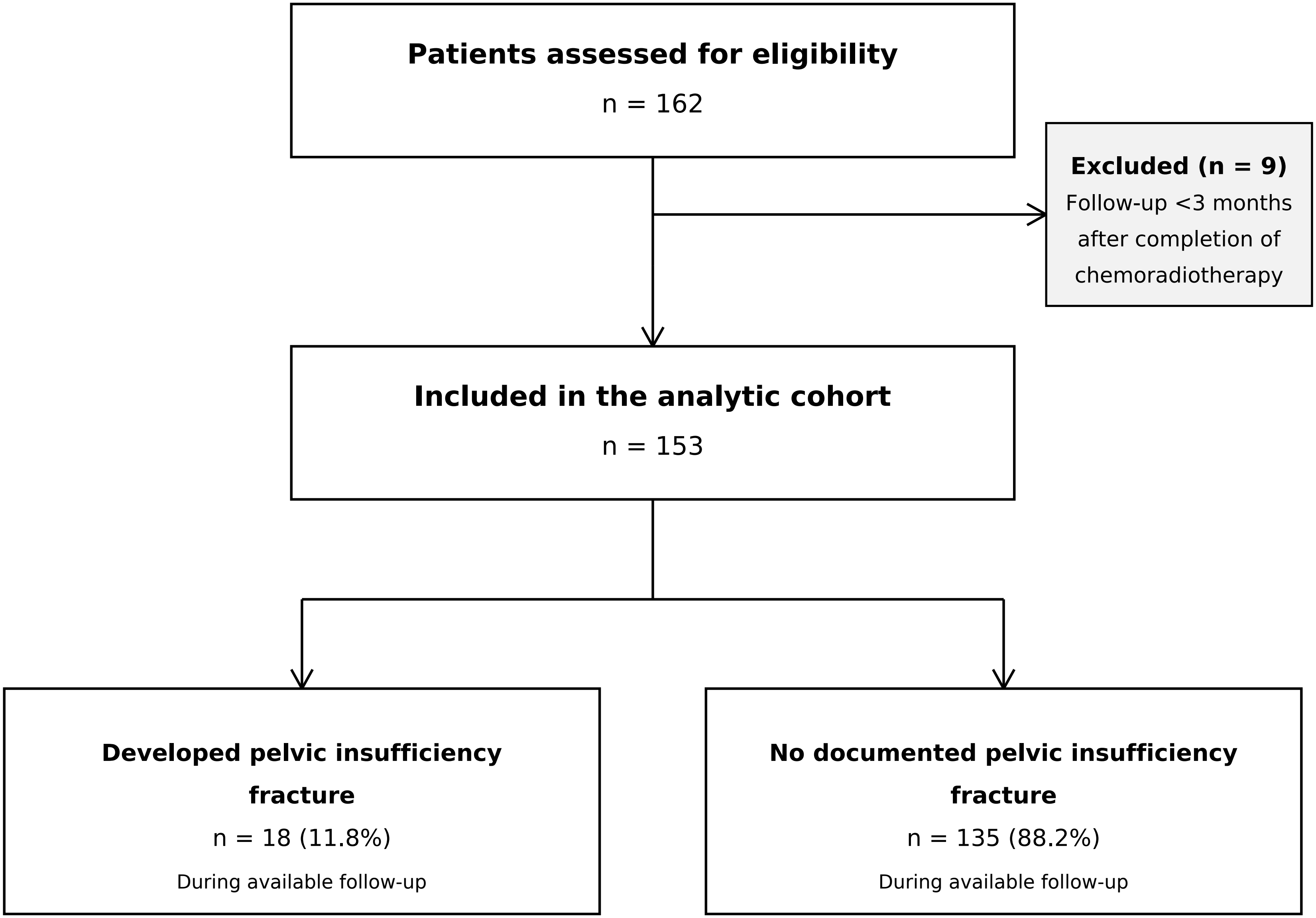

PIF, pelvic insufficiency fracture.
